# Carfilzomib resistance due to ABCB1/MDR1 overexpression is overcome by nelfinavir and lopinavir in multiple myeloma

**DOI:** 10.1038/leu.2017.212

**Published:** 2017-07-28

**Authors:** A Besse, S C Stolze, L Rasche, N Weinhold, G J Morgan, M Kraus, J Bader, H S Overkleeft, L Besse, C Driessen

**Affiliations:** 1 Experimental Oncology and Hematology, Department of Oncology and Hematology, Kantonsspital St Gallen, St Gallen, Switzerland; 2 Gorlaeus Laboratories, Leiden Institute of Chemistry and Netherlands Proteomics Centre, Leiden, The Netherlands; 3 Myeloma Institute, University of Arkansas for Medical Sciences, Little Rock, AR, USA

## Abstract

Proteasome inhibitor (PI) carfilzomib (CFZ) has activity superior to bortezomib (BTZ) and is increasingly incorporated in multiple myeloma (MM) frontline therapy and relapsed settings. Most MM patients ultimately experience PI-refractory disease, an unmet medical need with poorly understood biology and dismal outcome. Pharmacologic targeting of ABCB1 improved patient outcomes, including MM, but suffered from adverse drug effects and insufficient plasma concentrations. Proteomics analysis identified ABCB1 overexpression as the most significant change in CFZ-resistant MM cells. We addressed the functional role of ABCB1 overexpression in MM and observed significantly upregulated ABCB1 in peripheral blood malignant plasma cells (PCs) vs untreated patients’ bone marrow PC. ABCB1 overexpression reduces the proteasome-inhibiting activity of CFZ due to drug efflux, in contrast to BTZ. Likewise, the cytotoxicity of established anti-MM drugs was significantly reduced in ABCB1-expressing MM cells. In search for potential drugs targeting ABCB1 in clinical trials, we identified the HIV protease inhibitors nelfinavir (NFV) and lopinavir (LPV) as potent functional modulators of ABCB1-mediated drug export, most likely via modulation of mitochondria permeability transition pore. NFV and LPV restored CFZ activity at therapeutically relevant drug levels and thus represent ready-to-use drugs to be tested in clinical trials to target ABCB1 and to re-sensitize PC to established myeloma drugs, in particular CFZ.

## Introduction

Treatment regimens based on proteasome inhibitors (PIs) or immunomodulatory drugs are the current backbone of multiple myeloma (MM) therapy.^1^ Bortezomib (BTZ), the first-in class PI is a reversible, boronate-type inhibitor, whereas carfilzomib (CFZ), a second-generation PI, is an irreversible epoxyketone-based PI. The catalytic core of the proteasome contains three pairs of proteolytically active subunits with distinct substrate specificities (β1, β2 and β5; caspase-like, trypsin-like and chymotrypsin-like activity, respectively), out of which the β5 activity is rate limiting.^2^ Similar to BTZ, CFZ by design targets the β5 proteasome activity, albeit with a higher selectivity for the proteasome.^2^ Next-generation PI of the peptide boronate-type (ixazomib and delanzomib), the epoxyketone-type (oprozomib) and the β-lactone type (marizomib) share the same primary target and are in advanced development or already approved.^3^ CFZ has superior clinical anti-MM activity at a lower rate of neurotoxicity, compared with BTZ.^4^ After initial approval of CFZ for MM treatment in the relapsed/refractory setting, its approval advanced to second line therapy together with lenalidomide/dexamethasone and it is increasingly incorporated into frontline MM treatments in clinical trials.^4, 5^

MM treatment is usually not curative and most MM patients relapse after PI treatment or become PI refractory,^6, 7^ a condition with a very poor prognosis.^8^ The development of PI resistance under repetitive or continuing selective pressure with CFZ-containing regimens is an emerging clinical problem^9^ The biology of MM advances from intramedullary-restricted disease to extramedullary manifestations and finally to leukemia-like features with increasing proportions of malignant plasma cells (PCs) in the peripheral blood (PB). PB malignant PC represent the most aggressive state of MM cells and their number predict prognosis, with the worst outcome for overt PC leukemia (PCL).^10, 11, 12^

Proteasome inhibition-based MM therapy induces apoptosis through the induction of excessive proteotoxic stress.^13, 14^ Resistance of MM cells to PI involves concerted changes in cell maturation and metabolism.^15, 16^ However, PI resistance is not universal across the different classes of proteasome inhibiting drugs, ^16, 17^ suggesting drug-specific features.^16^ The biology of CFZ resistance in MM is poorly understood. CFZ-resistant MM cells show strong upregulation of ABCB1/P-gp, in contrast to BTZ-resistant MM.^16, 18, 19^

ABC (ATP-binding cassette) transporters, such as ABCB1 (multidrug resistance protein, MDR-1/P-glycoprotein and P-gp), ABCC1 (multidrug resistance-associated protein, MRP-1) and ABCG2 (breast cancer-related protein) mediate generic drug resistance of cancer by modulation of the absorption, disposition and elimination of xenobiotics and drugs.^20, 21^ ABCB1/P-gp is expressed on malignant PC in PB in MM^22, 23^ and is induced by chemotherapeutic agents, such as doxorubicin, in more than 50% of MM patients.^24, 25^ ABCB1 is the single most overexpressed protein when genetically matched CFZ-sensitive or resistant MM cell lines are compared by quantitative whole proteome profiling.^16^. Verapamil (VPM) as P-gp-inhibiting drug significantly improved MM treatment response in drug-resistant MM patients in the pre-PI era.^26, 27^ Although CFZ is a *bona fide* substrate for ABCB1,^18^ conflicting data exist for BTZ.^28, 29^ The functional role of ABCB1 overexpression for PI resistance of MM and its implications for the use of, or choice between, different PI drugs are unknown.

The HIV protease inhibitor (HIV-PI) drugs nelfinavir (NFV) and lopinavir (LPV) by design inhibit the HIV protease, a viral enzyme that lacks close homologies in eukaryotes.^30, 31^ NFV and LPV have been implicated in targeting antineoplastic pathways in human cancer cells in preclinical models,^32^ including AKT and the unfolded protein response, and were tested as sensitizing drugs in combination with radiation or chemotherapy in the clinic.^33, 34, 35^ Recently, the combination of oral NFV with standard dose BTZ/dexamethasone resulted in an 65% overall response rate in patients with heavily pretreated PI-refractory MM.^36^ HIV-PI have been suggested to be substrates of MDR proteins^37, 38^ but also to be MDR inhibitors.^39^

We here dissect the functional role of ABCB1 overexpression in CFZ-resistant MM and address the effect of ABCB1 on the cytotoxicity of standard MM drugs *in vitro*. We further identify NFV and LPV as off-the-shelve drugs able to decrease ABCB1 activity, to overcome CFZ resistance and to boost the activity of established drugs against advanced MM.

## Materials and methods

### Gene expression profiling and processing of patients’ data

Expression data of CD138+ PC from patients treated with Total Therapy regimens were processed as previously described.^40^ Detailed description of the Total Therapy regimens used is provided in Supplementary Table S1. Briefly, we used the Affymetrix U133 2.0 plus array custom (chip definition file) (v19) mapping to Entrez genes (http://brainarray.mhri.med.umich.edu/Brainarray/Database/CustomCDF/) as chip definition file. Expression data were normalized using GC-RMA. We excluded genes with log2 expression <3.5 in at least 95% of samples. Confining our analysis to autosomal genes expression data of 10,062 genes was available.

### Cell lines

The ARH77 PCL cell line and the AMO-1 MM cell line were obtained from commercial sources (American Type Culture Collection, Wesel, Germany and Deutsche Sammlung von Mikroorganismen und Zellkulturen, Braunschweig, Germany, respectively). Cells were maintained and adapted to BTZ (AMO-BTZ and ARH77-BTZ) and CFZ (AMO-CFZ and ARH77-CFZ) as previously described.^16, 41^

### Activity-based probes

Activity of proteasome subunits after PI treatment was assessed using the recently developed set of subunit-selective activity-based probes that differentially visualize individual activities of β1, β2 and β5 subunits of the constitutive and immunoproteasome.^42^

### Flow cytometry

Flow cytometric assessment of ABCB1 activity was performed using Mitotracker green FM (ThermoFisher Scientific, Waltham, MA, USA), a well-described substrate of ABCB1^(43, 44)^ and MVB003^(45)^ (a gift from Professor Overkleeft), an epoxyketone-based pan-reactive probe was used as ABCB1 substrate. Detailed and further description is provided in Supplementary Methods.

### CRISPR/Cas9 knockout of ABCB1

The two-vector CRISPR/Cas9 system was introduced into AMO-CFZ cells by lentiviral infection as described previously.^46, 47^ Details are specified in Supplementary Methods.

### Statistical evaluation

Statistical evaluation was performed in GraphPad Prism v.5 (GraphPad Software, La Jolla, CA, USA). For significance level two-tailed unpaired *t*-test was used, values *P*<0.05 were considered as statistically significant. If not indicated otherwise, results show one representative of at least three independent experiments.

## Results

### ABCB1 is consistently overexpressed in CFZ-resistant myeloma cell lines

**Figure 1:**
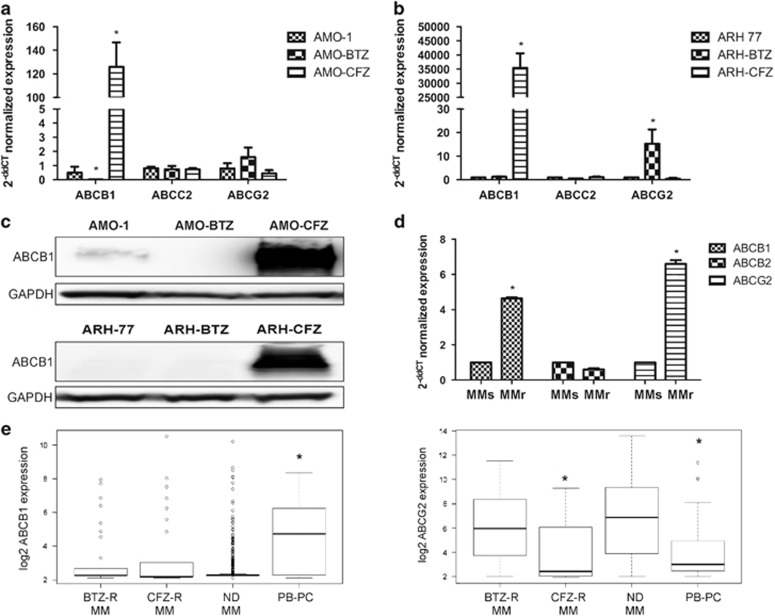
Expression of ABC-type transporters in cell lines and patients’ primary cells. (**a**) RNA expression of ABCB1, ABCC2 and ABCG2 transporter in AMO-1, AMO-BTZ and AMO-CFZ cell lines. (**b**) Expression of ABCB1, ABCC2 and ABCG2 transporter in ARH77, ARH-BTZ and ARH-CFZ cell lines. (**c**) Western blot validation of the overexpression of ABCB1 transporter in AMO-1 and ARH77 cell lines sensitive to PIs and their resistant counterparts. (**d**) Expression of ABCB1, ABCC2 and ABCG2 in primary patient’s sample: MMs, sensitive to CFZ; MMr, resistant to CFZ treatment. (**e**) Expression of ABCB1 and ABCG2 transporters in primary BTZ (BTZ-R) (*n*=33) and CFZ (CFZ-R) (*n*=29) resistant, newly diagnosed (NDMM) (*n*=1309) myeloma patients and in circulating PB–PC (*n*=44). Significant values <0.05 are marked with an asterisk (*).

Expression of the three major subfamilies of ABC-type transporters (ABCB1, ABCC2 and ABCG2) was assessed in AMO-1 and ARH77 cell lines, comparing the parental (PI-sensitive) cell lines (IC_50_ 4.8–6.3 nM) with their CFZ- or BTZ-resistant derivatives (AMO-BTZ, AMO-CFZ, IC_50_ 67–1342 nM) (Supplementary Table S2).^16, 41^ Expression of *ABCB1* mRNA was selectively upregulated in CFZ-resistant cells (Figures 1a and b), in contrast to BTZ-resistant cells, and concordant with protein expression (Figure 1c). Consistent with this, upregulation of *ABCB1*, but also *ABCG2*, was observed in a set of paired MM samples isolated from a MM patient initially responding to CFZ-based therapy and later progressing to PCL under such therapy (baseline sample taken before initiating CFZ-based therapy, second sample obtained during consecutive disease progression of the same patient under CFZ/dexamethasone therapy (Figure 1d)). Gene expression analysis before therapy from a patients cohort enrolled in ‘Total Therapy’ revealed that *ABCB1* was significantly increased in CD138+ circulating PB–PC of 44 newly diagnosed patients with primary PCL, compared with CD138+ bone marrow PC from 617 treatment-naive patients (Figure 1e). Further, we did not observe a difference in *ABCB1* expression between bone marrow PC from BTZ-refractory or CFZ-refractory patients and non-refractory newly diagnosed patients. *ABCG2* expression was significantly lower in PB–PC, compared with bone marrow PC from treatment-naïve or BTZ-refractory patients. We conclude that ABCB1 upregulation is a selective feature of circulating malignant PC from MM patients, which is associated with CFZ resistance *in vitro.*^16^

### Proteasome-inhibiting activity of CFZ, but not BTZ, is decreased in CFZ-resistant MM cells

**Figure 2:**
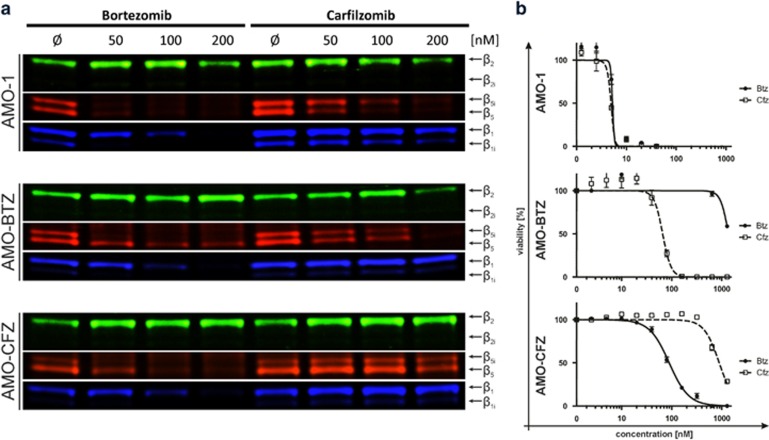
Comparison of dose–response of AMO-1 (sensitive) vs AMO-BTZ and AMO-CFZ (resistant) cells treated with PIs (BTZ and CFZ). (**a**) Residual activity of proteasome subunits visualized by activity-based probe labeling in intact cells after 1 h pulse treatment with indicated PIs. (**b**) Cell viability was measured after 48 hours of continuous treatment. Corresponding IC_50_ values are presented in Supplementary Table S2.

We next addressed whether ABCB1 overexpression in CFZ-resistant MM cells may lead to decreased cytotoxic activity due to impaired intracellular proteasome inhibition. We visualized proteasome activity of BTZ/CFZ-treated AMO-1, AMO-CFZ and AMO-BTZ cells using proteasome selective activity-based chemical probes, and at the same time assessed cell viability (Figures 2a and b). With BTZ treatment, we observed dose-dependent inhibition of β1 proteasome activity of very similar efficacy in all three cell types, as expected. BTZ likewise resulted in near-complete β5 inhibition in AMO-1 and AMO-CFZ, whereas β5 inhibition was less effective in AMO-BTZ, consistent with the β5 active-site mutation present in these cells.^16^ CFZ treatment led to very similar dose-dependent inhibition of β5 activity in AMO-1 and AMO-BTZ, and at high concentrations also β2 activity, as expected. However, in AMO-CFZ, CFZ up to 100 nM lacked a detectable inhibitory effect on intracellular β5 or β2 proteasome activity. This corresponded to differential cytotoxic sensitivity of AMO-CFZ and AMO-BTZ against their respective selecting drugs (BTZ or CFZ): AMO-CFZ had an ~10-fold higher IC_50_ for CFZ than for BTZ treatment and *vice versa*, and AMO-BTZ showed a 20-fold higher IC_50_ for BTZ, compared with CFZ (Figures 2a and b, and Supplementary Table S2).

**Figure 3:**
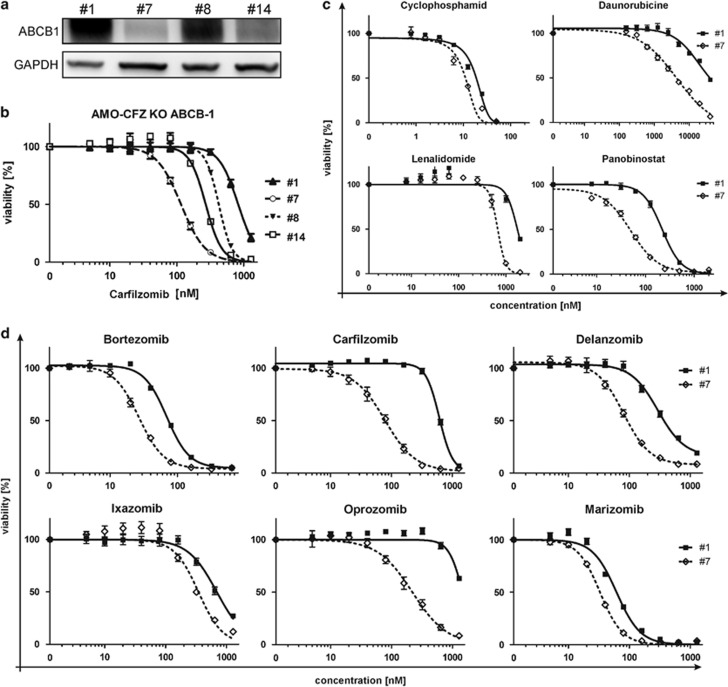
Impact of ABCB1 knockdown on the sensitivity of AMO-1 cells. (**a**) Western blot evaluation of the ABCB1 depletion in AMO-CFZ clones obtained by CRISPR/Cas9 genome editing tool. (**b**) Dose–response of AMO-CFZ clones with depleted ABCB1 to CFZ. Corresponding IC_50_ values are presented in Supplementary Table S3. (**c**) Dose–response of selected AMO-CFZ clones 1 and 7 to CFZ and convectional drugs used in the treatment of MM. (**d**) Dose–response of AMO-CFZ clones 1 and 7 to approved PI or those in clinical development. Corresponding IC_50_ values are presented in Supplementary Table S4.

To directly address the functional role of ABCB1 for CFZ resistance, we eliminated ABCB1 protein in AMO-CFZ cells by CRISPR/Cas9 (for example, clone 7) (Figure 3a). We observed an inverse correlation between the levels of ABCB1 and myeloma cell sensitivity to CFZ (Figure 3b, Supplementary Table S3). Subtotal elimination of ABCB1 resulted in an approximately eightfold decrease of the IC_50_ for CFZ. Together, these data demonstrate that ABCB1-mediated export of CFZ limits CFZ-induced cytotoxicity in CFZ-resistant MM. Overexpressed ABCB1 is therefore a therapeutic target for CFZ-resistant myeloma.

### ABCB1 overexpression interferes with activity of approved myeloma drugs

To dissect the role of ABCB1 overexpression for the activity of standard myeloma drugs we compared the cytotoxic effects of such drugs on AMO-CFZ cells with (clone #1) or without such ABCB1 expression (clone 7) (Figure 3c and Supplementary Table S4). To score for good versus poor ABCB1-substrate myeloma drugs, we calculated the ratio of IC_50_ values between the ABCB1-containing and the ABCB1-deficient clone for each of the myeloma drug. For the known ABCB1 substrate daunorubicin, this ratio was 4.8, whereas it was 1.6 for cyclophosphamide, suggesting that cyclophosphamide is almost ABCB1 independent. Interestingly, the epoxyketone-type proteasome inhibiting drugs, (oprozomib and CFZ) scored even higher than daunorubicin in this comparison (ratio of 6.7 and 7.8, respectively). Likewise, panobinostat and delanzomib were considerably strong ABCB1 substrates, almost comparable to daunorubicin, with ratios of 4.4 and 3.7, respectively, whereas BTZ, lenalidomide and ixazomib showed moderately low degrees of ABCB1 interaction with scores around 2.6, 2.6 and 2, respectively, and marizomib (score of 1.6) showed the weakest interaction with ABCB1. The data demonstrate that cytotoxicity of the epoxyketones CFZ and oprozomib is very sensitive to ABCB1 overexpression, whereas anti-MM activity of marizomib is almost independent from level of ABCB1.

### NFV and LPV strongly decrease ABCB1 activity

**Figure 4:**
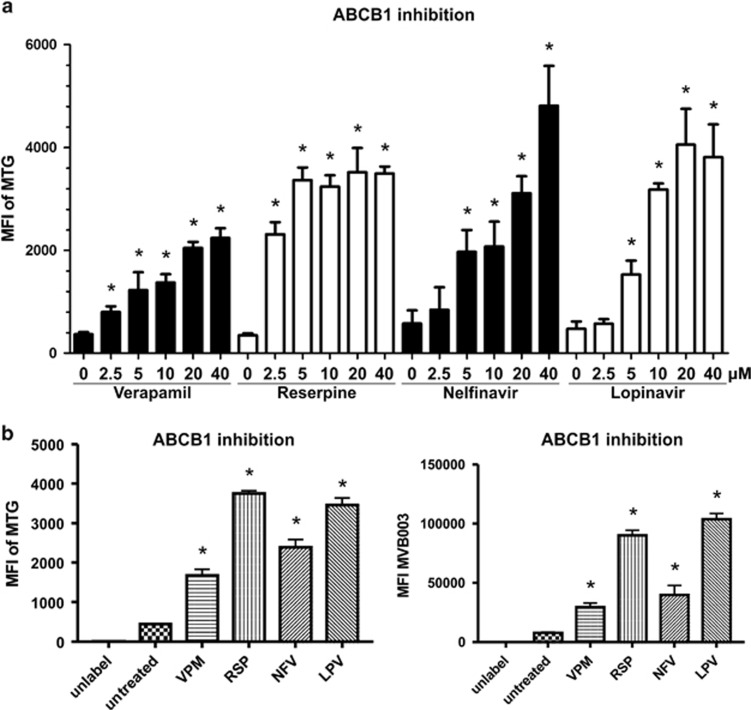
Functional inhibition of ABCB1 by VPM, RSP, NFV and LPV evaluated by Mitotracker Green FM and MVB003 efflux. (**a**) Dose–response inhibition of ABCB1 evaluated by Mitotracker Green FM (MTG) efflux after 12 h of treatment with indicated concentrations of compounds. (**b**) Inhibition of ABCB1 after 12 h of treatment with 10 μM concentration of VPM, RSP, NFV and LPV evaluated by MTG and MVB003 efflux. Significant values <0.05 are marked with an asterisk (*).

P-gp inhibition can functionally be assessed by measuring the intracellular accumulation of fluorescent dyes like Mitotracker Green FM, which is exported from the cytoplasm by functional P-gp.^18^ To exclude a possible interference of the dye with mitochondria, a modified epoxyketone-based PI tagged with fluorescent probe, MVB003, was used. Culture of AMO-CFZ in the presence of Mitotracker Green FM or MVB003 resulted in only a weak fluorescence signal from intracellular dye, consistent with overexpression of ABCB1 and ABCB1-mediated export of the dye. Addition of VPM or reserpine (RSP) in the micromolar range led to a sizable, dose-dependent increase in cellular fluorescence in AMO-CFZ, as expected, consistent with the inhibition of P-gp mediated export by the drugs (Figures 4a and b). A very similar effect was observed when cells were co-incubated with the HIV-PI NFV and LPV (Figures 4a and b), suggesting that NFV and LPV likewise modulate P-gp in AMO-CFZ. NFV and LPV functionally decreased P-gp activity already at drug concentrations between 5 and 10 μM already after 1 h incubation, matching the serum NFV/LPV concentrations achieved in patients (Figure 4a and Supplementary Figure S1).^48^

### P-gp inhibition increases activity of CFZ in CFZ-resistant myeloma

**Figure 5:**
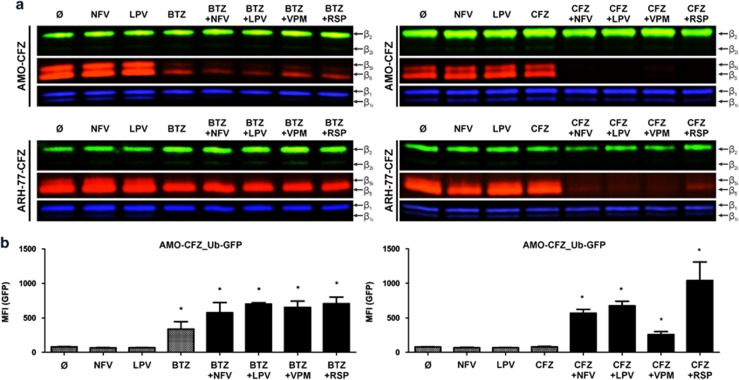
Impact of VPM, RSP NFV and LPV on the CFZ entrance into the AMO-CFZ cells. (**a**) Residual activity of proteasome subunits visualized by activity-based probe labeling after 12 h pre-treatment with indicated compounds and subsequent 1 h treatment with 50 nM CFZ. (**b**) AMO-CFZ stably expressing Ub-GFP co-treated for 12 h with 10 μM concentration of indicated compounds in combination with 50 nM CFZ. Significant values <0.05 are marked with an asterisk (*).

We used activity-based probe to demonstrate that P-gp-inhibiting drugs re-establish the intracellular proteasome-inhibiting activity of CFZ in CFZ-resistant MM cells. AMO-CFZ and ARH-77-CFZ cells showed adequate reduction in intracellular proteasome activity upon pulse treatment with BTZ (25 nM, 1 h) compared with untreated cells or NFV/LPV controls and proteasome activity was not affected by co-treatment with VPM, RSP, NFV or LPV (10 μM) in this setting. Proteasome activity was not affected when AMO-CFZ and ARH-77-CFZ were treated with CFZ (50 nM, 1 h), consistent with the results above. Strikingly, treatment with CFZ and the MDR-1 inhibitors VPM, RSP, NFV or LPV (10 μM) restored the full proteasome-inhibiting activity of CFZ (Figure 5a). We next addressed whether such increase in intracellular proteasome inhibition by co-administration of ABCB1-inhibiting drugs would also functionally translate into increased intracellular accumulation of proteasome substrate protein upon BTZ/ CFZ challenge. AMO-CFZ were equipped with stable expression of GFP-modified ubiquitin (Ub^G67V^-GFP), which is incorporated in the poly-ubiquitin chain to identify and quantify proteasome substrate proteins, so that an increase in fluorescence signifies the intracellular accumulation of proteasome substrate proteins.^49^ In untreated AMO-CFZ_Ub-GFP cells or respective cells treated with NFV or LPV alone, no significant fluorescence signal was detected, indicating undisturbed proteasomal proteolysis. Treatment with 25 nM BTZ resulted in significant fluorescence and thus functionally relevant proteasome inhibition. This was further increased in the presence of VPM, RSP, NFV or LPV. CFZ treatment (50 nM 1 h pulse) alone did not result in an Ub-GFP-fluorescence signal, whereas its combination with VPM, RSP, NFV or LPV again re-established functional proteasome inhibition. Interestingly, of the clinically available drugs, the synergistic effect of NFV and LPV with CFZ was considerably stronger than VPM at the same concentration (Figure 5b). Taken together, the data demonstrate that CFZ is a strong substrate for ABCB1 and that MDR-blocking drugs can be used to re-establish the proteasome-inhibiting activity of BTZ/CFZ in CFZ-resistant MM cells. They further identify NFV and LPV as established drugs that mediate this effect at low micromolar concentrations.

### Mapping the ABCB1-modulating portion of NFV and mechanism of P-gp inhibition by NFV and LPV

**Figure 6:**
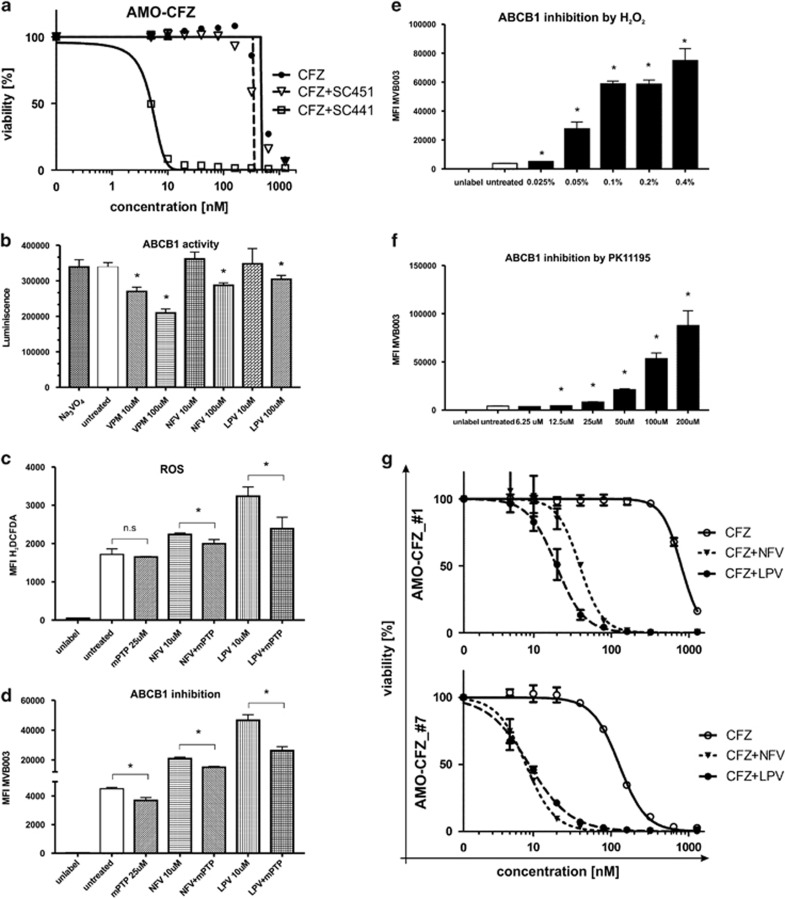
Evaluation of the specificity of NFV and LPV in the modulation of ABCB1 function and importance of ABCB1 in the CFZ resistance. (**a**) Dose–response of AMO-CFZ resistant cells to CFZ and co-treatment with SC451 and SC441. Corresponding IC_50_ values are presented in Supplementary Table S5. (**b**) Activity of ABCB1 evaluated by PgP-Glo assay. (**c**) Rescue experiment of ROS induction in AMO-CFZ after treatment with mPTP inhibitor (decylubiqinone), NFV, LPV and combination evaluated by H_2_DCFDA staining. (**d**) Rescue experiment of functional inhibition of ABCB1 in AMO-CFZ evaluated by MVB003 efflux after treatment with mPTP inhibitor (decylubiqinone), NFV, LPV and combination. (**e**) Functional inhibition of ABCB1 in AMO-CFZ evaluated by MVB003 efflux after 30 min treatment with increasing concentration of H2O2. (**f**) Functional decrease of ABCB1 function in AMO-CFZ evaluated by MVB003 efflux after 12 h treatment with increasing concentration of PK11195, mPTP facilitator. (**g**) Dose–response of AMO-CFZ_ABCB1 high (1) and low (7) with or without 10 μM NFV and 10 μM LPV co-treatment. Corresponding IC_50_ values are presented in Supplementary Table S6. Significant values <0.05 are marked with an asterisk (*).

HIV-PI by design inhibit the HIV protease, which lacks close human homologues. To map the ABCB1-inhibiting activity of NFV to a functional region of the drug molecule, we synthesized two analogues of NFV: SC451, a non-functional derivative where the predicted target interaction site is occupied by an acyl group, and compared its ABCB1-inhibiting activity to SC441, a structural analog of NFV (Supplementary Figure S2). Although SC441 completely retained the CFZ-sensitizing activity of the NFV parent drug, SC451 was inactive (Figure 6a and Supplementary Table S5). This maps the ABCB1-inhibiting activity of NFV to the region of SC451 occupied by the acyl group.

To reveal the underlying mechanism of ABCB1 modulation by NFV and LPV, we performed PgP-Glo assay, which serves to elucidate whether NFV and LPV are direct ABCB1 inhibitors or substrates. NFV and LPV were confirmed to be neither direct inhibitors, nor strong ABCB1 substrates, similar to VPM (Figure 6b). Therefore, we focused on the modulation of mitochondrial function as an indirect mechanism of functional ABCB1 inhibition. Indeed, NFV was shown to modulate mitochondria the activity of the mitochondrial permeability transition pore (mPTP),^50^ and NFV and LPV induce reactive oxygen species formation, which likely mirrors a modulation of mitochondrila activity.^51, 52^ Decylubiquinone, a known mPTP inhibitor antagonizes reactive oxygen species production and likewise counteracted ABCB1 inhibition induced by NFV or LPV (Figures 6c and d). On contrary, mPTP facilitators (H_2_O_2_ or PK11195)^53^ induced dose-dependent inhibition of ABCB1 function, leading to increased CFZ-mediated cytotoxicity in AMO-CFZ (Figures 6e and f, Supplementary Figure S3 and Supplementary Table S8). Thus, the data support that mPTP function is activated by NFV and LPV, which in turn is directly linked to ABCB1 inhibition.

As the mechanism of action of NFV to overcome PI resistance likely involves additional mechanisms besides ABCB1 inhibition, we aimed to establish the specific contribution of the ABCB1-mediated vs ABCB1-independent role of NFV or LPV against MM. In the presence of functional ABCB1, LPV or NFV treatment resulted in a 20–40-fold decrease of IC_50_ for CFZ. In the absence of ABCB1, LPV and NFV retained sizable CFZ-sensitizing activity, but the decrease in the IC_50_ was only 13–15-fold. A very similar pattern was also observed for VPM and RSP co-treatment (16–60-fold and 11–13-fold, respectively) (Figure 6g and Supplementary Tables S6A and B). This is consistent with a functionally important role of ABCB1-mediated export of CFZ in AMO-CFZ cells, which is reduced by NFV or LPV. It further demonstrates that LPV and NFV target additional molecules that substantially contribute to drug resistance.

### NFV or LPV have superior PI-sensitizing activity in combination with epoxyketone-type PIs

**Figure 7:**
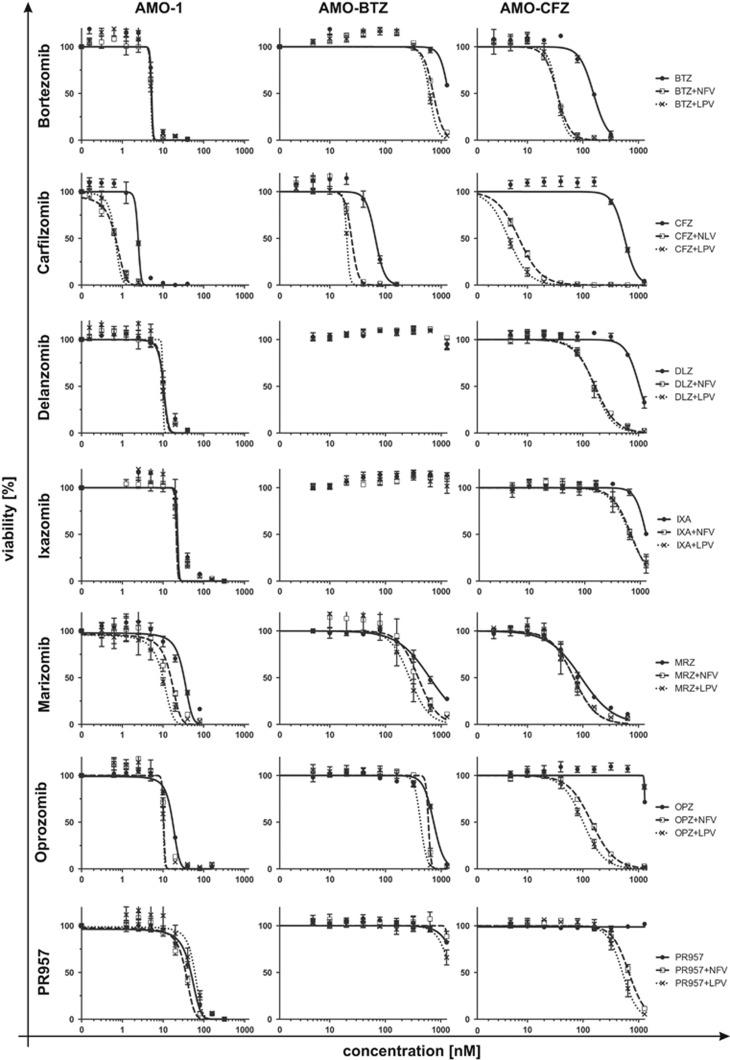
Sensitivity profile of ABCB1 inhibitors co-treatment with PIs used in the clinic or in clinical development in AMO-1, AMO-BTZ and AMO-CFZ cell lines. Dose–response curves of PI co-treated with NFV (10 μM) and LPV (10 μM). Corresponding IC_50_ values are presented in Supplementary Table S7.

To address to what extent NFV or LPV likewise sensitizes myeloma cells to next-generation PIs, we established the IC_50_ for the approved proteasome inhibiting drugs or drug candidates: BTZ, ixazomib, delanzomib (peptide boronate-based), CFZ, oprozomib, the immunoproteasome-selective PR957 (epoxyketone) and the β-lactone marizomib, in the presence/absence of NFV or LPV by viability assays. The highest PI-sensitizing effect of NFV/LPV (10–120-fold, Supplementary Table S7) was observed when ABCB1-overexpressing cells (AMO-CFZ) were treated with the strong ABCB1 substrates, such as the epoxyketone-type of inhibitors CFZ or oprozomib (Figure 7). In cells with low ABCB1 expression (AMO-BTZ), NFV and LPV likewise increased the sensitivity of PI-resistant cells for epoxyketone-type PI, although the effect was considerably weaker compared with ABCB1-overexpressing cells (AMO-CFZ). An exception here was marizomib, which showed strong drug-sensitizing effect by NFV/LPV in AMO-1 cells without features of adaptive resistance. These data characterize LPV and NFV as powerful drugs to increase PI sensitivity of MM cells towards the entire spectrum of proteasome inhibitory drugs or drug candidates. LPV and NFV are especially effective in the CFZ-resistant setting and/or in combination with CFZ.

## Discussion

We demonstrate that ABCB1 is overexpressed on circulating PB malignant PC in newly diagnosed PCL patients, and that ABCB1 overexpression results in CFZ resistance via ABCB1-mediated export of CFZ. However, we did not observe increase of *ABCB1* expression in PC of CFZ-refractory patients. It has been previously shown that PC loose P-gp function when they home to the bone marrow and re-activate it again while leaving the marrow.^23^ Further, it has been shown that malignant MM cell population of patients relapsing under PI treatment consist of multiple subclones with different maturation stages within each subclone, from CD138+,CD38+ to CD138−,CD38−,CD20+ MM cells;^15^ therefore, gene expression profiling analysis of CD138+ cells is likely not representative of the whole PI-resistant population. Nevertheless, we show here that circulating PC are *de-novo* expressing ABCB1. ABCB1 expression is likely a feature that is rather specific for B-cells and circulating PC. Its expression has been shown to correlate with poor prognosis, treatment resistance and aggressive disease.^22, 54, 55, 56^ Relevance of ABCB1 expression in MM is supported by its strong, consistent and selective upregulation in several CFZ-resistant cell lines, as well as in a malignant PC patient sample that was analyzed before and after acquisition of CFZ resistance. The *in vitro* model of CFZ-adapted MM cells used here matches the currently known major characteristics of PI-refractory MM, such as lack of proteasome mutation and IRE1/XBP1-low stage.^15, 16^ Overexpression of the ABCB1 drug exporter has been observed in MM cells, including CFZ-resistant MM,^16^ but its functional significance was unclear. ABCB1 overexpression is predicted to result in P-gp-mediated drug export. We directly demonstrate that increased ABCB1 limits the proteasome-inhibiting activity and clearance of poly-ubiquitinated protein by CFZ in CFZ-resistant malignant PC overexpressing *ABCB1*, which results in significantly reduced cytotoxicity.

Our data implicate ABCB1 as a therapeutic target and a modulator of the efficacy of MM drugs in relapsed and highly advanced MM and particularly in the CFZ-exposed setting. The current therapeutic trend to place CFZ earlier in the MM treatment algorithm and to foresee prolonged CFZ treatment periods may contribute to an increased emergence of ABCB1-overexpressing MM clones in the future. ABCB1 overexpression affected the cytotoxic activity of epoxyketone-type PIs significantly stronger than non-epoxyketone PIs. Other than toxicity issues, there is currently little rationale to use a particular class of PIs vs another class to achieve the best possible anti-MM activity in the clinic. Our data show that epoxyketone-type PIs are less active in ABCB1-overexpressing MM, suggesting that ABCB1-overexpressing patients may rather benefit from other classes of PIs, in particular marizomib or ixazomib. We also show that *in vitro* adaptation to BTZ results in ~10-fold higher sensitivity to CFZ than to BTZ, whereas the opposite is true when MM cells adapt to CFZ. Given that most patients included in the ENDEAVOR trial were BTZ-pretreated (and a significant number even BTZ refractory), but almost no patients were CFZ-pretreated, BTZ-pretreatment may likewise have skewed the results towards favoring CFZ sensitivity in the ENDEAVOR trial.^4^ This possibility may be taken into account when interpreting the outcome of this head to head comparison.

Our results further suggest that ABCB1-overexpressing MM may likewise have a low sensitivity to other strong ABCB1 substrate drugs used in MM therapy, such as panobinostat or daunorubicin, but may be better targeted with ixazomib, cyclophosphamide or marizomib, which lack significant ABCB1 interaction. Of the established MM drugs, we identified cyclophosphamide as the one with the least sensitivity to ABCB1-mediated export. This matches well with the striking activity observed when cyclophosphamide was added to a pomalidomide-dexamethasone backbone in patients with heavily pretreated MM, of which 44% were CFZ refractory.^9^ The low sensitivity of cyclophosphamide to ABCB1-mediated export could have significantly contributed to the therapeutic activity of cyclophosphamide in this setting in heavily pretreated MM patients.

We here identify NFV and LPV, two approved HIV-PI, as potent drugs preventing ABCB1-mediated export of CFZ by MM cells. NFV and LPV overcome CFZ resistance at low micromolar concentrations. Appropriate plasma levels of NFV (~10–15 μM) have been consistently reached and tolerated well in a recent phase I trial, where we tested escalating doses of NFV in combination with full dose PI therapy in patients with advanced hematologic malignancies, including PI-refractory MM.^57^ A respective national phase II trial in BTZ-refractory MM achieved an 65% overall response rate in heavily pretreated patients with PI-refractory MM, an unprecedented response rate in this category of patients achieved with registered drugs.^36^ Swissmedic has granted orphan drug status to NFV for MM treatment.

Previous attempts to establish P-gp inhibiting drugs as chemotherapy sensitizers in MM have failed due to undesirable pharmacokinetic interactions of the drugs used, their inability to achieve sufficiently high plasma concentrations and the lack of a diagnostic setup to safely identify patients with P-gp overexpressing MM.^58^ NFV and LPV are off-the-shelf drugs that overcome these limitations and may be tested in clinical trials. The use of P-gp-sensitive intracellular dyes in conjunction with flow cytometry allows functional testing of patient MM cells for P-gp activity in a broadly applicable fashion. This may allow establishing a valid biomarker for MM patients that are likely to benefit from P-gp targeting drugs, like NFV or LPV. NFV and LPV act synergistically with all known and future PI against MM *in vitro* (Figure 7). In addition, they decrease ABCB1-mediated drug efflux that limits the activity of non-PI MM drugs such as lenalidomide, daunorubicine or panobinostat (Figure 3). Thus, NFV and LPV have a very broad drug-sensitizing potential for MM therapy.

The molecular target of NFV in MM is still unknown. High doses of NFV have been suggested to directly inhibit proteasome function, but we have previously shown that NFV and LPV do not impair proteasome activity at drug concentrations <40 μM and likewise, NFV at drug-sensitizing concentrations of 10 μM did neither affect proteasome subunit activity nor the accumulation of polyubiquitinated proteasome substrate protein in our results (Figure 5). We have also previously shown that NFV activates unfolded protein response in PI-sensitive and PI-resistant cells, which results in increased BTZ-sensitivity *in vitro* and is associated with BTZ-sensitivity in the clinic.^59^ Here we further show that NFV and LPV inhibit the ABCB1 efflux by facilitating the mPTP activity, consistent with previous reports suggesting that NFV modifies mitochondria function by mPTP modulation.^50^ Our data obtained with/without genetic ablation of ABCB1 in ABCB1-overexpressing AMO-CFZ cells provide further proof that NFV and LPV act at least in part through P-gp inhibition, in particular in cells with ABCB1 overexpression. The synergy between CFZ and NVF/LPV is substantially higher than that seen for BTZ. However, the >1 log difference in IC_50_ between AMO-CFZ lacking ABCB1 expression and treated with CFZ in the presence or absence of NFV clearly demonstrates that additional molecular targets mediate the PI-sensitizing activity of NFV. It is likely to be that NFV and LPV block the activity of additional multidrug resistance proteins in a similar manner. This is supported by the substantially larger accumulation of Ub^G67V^-GFP-labeled proteasome substrate proteins in CFZ-resistant cells co-treated with RSP, NFV or LPV, compared with VPM at the same dose (Figure 5). Although VPM is a rather selective inhibitor of P-gp, RSP has a wider substrate selectivity that includes several other multidrug resistance proteins.^60^ However, NFV and LPV also significantly increased the sensitivity of BTZ-resistant cells (which do not express MDR-type proteins) against marizomib (PI with the lowest interaction potential with ABCB1) and BTZ, suggesting that NFV/LPV act through additional cellular targets beyond MDR-type drug export proteins. Importantly, we were able to map this activity to a defined region within the NFV compound. This truncated version of NFV may now be used as a point of departure to identify the additional molecular target(s) and to generate novel lead compounds against MM by medical chemistry approaches aiming at substantially lower IC_50_ values.

In summary, we here demonstrate the functional importance of ABCB1 overexpression and P-gp-mediated export of therapeutic drugs, including CFZ, in CFZ-resistant MM. We further identify NFV and LPV as ready-to-use, approved drugs that modulate P-gp function and overcome P-gp-mediated drug resistance in MM, presumably by an indirect mechanism that involves mPTP. NFV and LPV substantially increase the activity of several myeloma drugs in this setting at concentrations that are clinically tolerable and achievable. Our results open immediate options to exploit these activities in clinical trials in advanced MM.
