# Supplementary material for: Carfilzomib resistance due to ABCB1/MDR1 overexpression is overcome by nelfinavir and lopinavir in multiple myeloma
Source: Leukemia. 2017 Jul 28;32(2):391–401. doi: 10.1038/leu.2017.212 (PMC5808083; doi:10.1038/leu.2017.212)
Supplement: Supplementary Information [file leu2017212x1.docx]

**SUPPLEMENTAL DATA**

**for the manuscript of Besse et al.: Carfilzomib resistance due to ABCB1/MDR1 overexpression is overcome by nelfinavir and lopinavir in multiple myeloma**

**SUPPLEMENTAL MATERIAL AND METHODS**

**Patients primary material used in this study**

Primary cells were obtained from peripheral blood of patient with multiple myeloma progressing to plasma cell leukemia during routine diagnostic procedures after approval by the independent cantonal ethical committee and after obtaining written informed consent form. Primary cells were enriched by Ficoll density gradient centrifugation. Primary cell preparations were analyzed microscopically after routine staining and only preparations with > 80% malignant cells were used for experiments described here.

**Chemicals used**

The following compounds were used in the work: marizomib (NPI-0052, Adipogen, Switzerland), delanzomib (CEP-18700, Selleckchem, TX, USA), oprozomib (ONX0912, Selleckchem, TX, USA), ixazomib (MLN9708, Selleckchem, TX, USA) HIV inhibitors nelfinavir (NIH AIDS research, USA) and lopinavir (NIH AIDS research, USA), lenalidomide (Celgene Corporation, USA), ABCB1 inhibitors verapamil (Sigma-Aldrich, MO, USA) and reserpine (Sigma-Aldrich, MO, USA), daunorubicin (Calbiochem/EMD Milipore, MA, USA), panobinostat (Selleckchem, TX, USA), cyclophosphamide (Sigma-Aldrich, MO, USA), decylubiquinone (#D7911; Sigma-Aldrich, MO, USA), PK11195 (Sigma-Aldrich, MO, USA), Hydrogen peroxide solution (H2O2; Sigma-Aldrich, MO, USA).The proteasome inhibitors bortezomib, carfilzomib, PR957 (β5i specific) and analogues of nelfinavir (non-functional SC451, functional SC441) were synthesized at the Leiden Institute of Chemistry.

**CRISPR/Cas9 knockout of ABCB1**

Lentivirus was produced by packaging plasmids pMD2.G and psPAX2 (a gift from Trono’s lab; Addgene plasmids #12259 and #12260) and transfer plasmids lentiCas9-Blast or lentiGuide-Puro (a gift from Zhang’s lab; Addgene plasmids #52962 and #52963). This two-vector system allows delivery of Cas9 and sgRNA on separate viral vectors with distinct antibiotic selection. After Cas9 infection cells were selected by Blasticidine S (Sigma-Aldrich, MO, USA), cells with stably introduced Cas9 were infected with particles containing sgRNA targeting ABCB1, exon 2 (sgRNA sequence was designed using online tool: crispr.mit.edu; sgRNA for ABCB1: *CCT*GAGCTCATTCGAGTAGCGGC) and selected by Puromycin (Sigma-Aldrich, MO, USA). Cells were subcloned, clones screened for the ABCB1 mutation by T7E1 assay (New England Biolabs, MA, USA), and tested for ABCB1 protein knockdown by western blot. Sanger sequencing was performed to confirm the presence of a mutation in a desired part of genome using forward primer for ABCB1/ exon 2: 5’-GGAGCAGTCATCTGTGGTGAG-3’.

**Generation of AMO-CFZ Ub-G67V-GFP cells**

AMO-CFZ cells were electroporated with a plasmid containing Ub-G76V-GFP [[1](#_ENREF_1)] (obtained from Nico Dantuma; Addgene plasmid #11941) and subsequently cultured in the presence of selecting antibiotic G418 (500ng/ml, Gibco/Invitrogen, MA, USA). Subclones were obtained using MethoCult (StemCell Technologies, USA) and the clone with highest accumulation of fluorescence after BTZ treatment was chosen for further analysis.

**Assessment of cell viability**

Viability of cell lines was determined after 48h of treatment by MTS tetrazolium compound using CellTiter 96® AQueous One Solution) (Promega, WI, USA) according to manufactures protocol.

**PgP-Glo assay**

PgP-Glo assay (Promega, WI, USA) was performed according to manufacturer recommendations.

**Western blotting**

SDS-PAGE and Western blot was performed on precast 12% gels as described [[2](#_ENREF_2)], using the following antibodies: anti-MDR1/ABCB1 (E1Y7S; rabbit mAb #13978; Cell Signaling Technology, MA, USA), anti-GAPDH-HRP conjugate (#hrp-60004; Proteintech, IL, USA).

**Flow cytometry**

Cells were seeded as 3x10^5^/ml and subsequently treated with 10 µM verapamil (VPM), reserpine (RSP), nelfinavir (NFV), lopinavir (LPV). PK11195, H2O2 and decylubiquinone concentrations are specified in a relevant section.

For functional analysis of ABCB1 inhibition, cells were incubated with the compounds for 12h before MTG (100 nM final concentration) or MVB003 (1 µM final concentration) was added for a 20 min/ 37°C or 30 min/ 37°C incubation, respectively, followed by washing with PBS and analysis by flow cytometer (BD FACS Canto II and BD Fortessa; BD Biosciences, USA). For the estimation of GFP fluorescence in AMO-CFZ-Ub-G76V-GFP after treatment, cells were treated as described above for 8h and GFP fluorescence was acquired by flow cytometer (BD FACS Canto II BD Biosciences, USA).

# For the measurement of intracellular ROS levels, cells were incubated with 10 μM 2′,7′-Dichlorofluorescin diacetate (H_2_DCFDA; Sigma-Aldrich, MO, USA) for 20 min at 37°C in the dark. Cells were washed, harvested and green fluorescence intensity was examined by FACS Canto II (BD Biosciences, CA, USA). Data were evaluated using FlowJo v10 Software (FlowJo Company, Ashland, OR, USA) and are presented as a mean and ±SD of median fluorescence intensity (MFI) of at least 3 independent experiments.

**Quantitative PCR**

Total RNA was isolated using Direct-zol RNA MiniPrep kit (Zymo research, CA, USA) and Trizol (Ambion/Thermo Fisher Scientific, MA, USA) and reversely transcribed into cDNA using High Capacity cDNA Reverse Transcription Kit (Applied Biosystems/ Thermo Fisher Scientific, MA, USA). QPCR was performed in duplex reaction with 10ng of cDNA using 2XTaqMan Gene Expression Master Mix, TaqMan specific assays for ABCB1, ABCC2 and ABCG2 (Hs00184500_m1; Hs00166123_m1 and Hs01053790_m1) and GAPDH as endogenous control (#4326317E; all Applied Biosystems/Thermo Fisher Scientific, MA, USA) according to manufacturer’s recommendations on Light Cycler II (Roche, Switzerland). Quantitative PCR was performed from total RNA after reverse transcription in a duplex reaction using a commercial system with GAPDH endogenous control.

**Chemical synthesis**

**General synthetic methods**

All reagents used were of commercial grade and used as received. Tetrahydrofuran (THF), dichloromethane (DCM) and N,N-dimethylformamide (DMF) were dried over activated 4 Å molecular sieves; methanol (MeOH) was dried over 3 Å molecular sieves prior to use. All other solvents were of p.a. quality. Column chromatography was performed using Screening Devices b.v. silica gel with a particle size of 40-63 µm and a pore diameter of 60 Å. TLC analysis was carried out using Merck pre-coated aluminium sheets (silica gel 60, F254) and detection by UV absorption and spraying with a solution of KMnO_4_ (20 g/L) and NaOH (10 g/L) followed by charring at ca. 150 °C. ^1^H and ^13^C spectra were recorded on a Bruker AV-500 (500 MHz) or AV-600 (600 MHz) spectrometer. Chemical shifts are given in ppm (δ) relative to the residual deuterated solvent. Coupling constants (*J*) are given in Hz. High resolution mass spectra were recorded on a LTQ Orbitrap (Thermo Finnigan, San Jose, CA, USA) equipped with an electrospray ion source. LC-MS analysis was performed on a Surveyor HPLC system (Thermo Finnigan, San Jose, CA, USA) equipped with a C18 column (Gemini, 4.6 mm x 50 mm, 3.0 µm particle size, Phenomenex) coupled to an LCQ Advantage Max (Thermo Finnigan, San Jose, CA, USA) ion trap spectrometer (ESI). The buffers applied were A: H_2_O, B: acetonitrile (MeCN) and C: 1% aqueous trifluoroacetic acid (TFA). Reversed-phase HPLC purifications were carried out on a Waters autopurification system equipped with an SQ Mass Detector and a continuous UV detector (200-600 nm) using a preparative Phenomenex Gemini C18 (21 x 150 mm) column. The buffers applied were A: H_2_O + 0.2% TFA, B: MeCN.

**Synthesis of nelfinavir probes SC441 and SC451**





**Extraction of nelfinavir from Viracept tablets (nelfinavir mesylate)**

Viracept tablets (4x 625 mg) were crushed carefully and the coating was removed before slurring the powder in 10% (w/v) aqueous NaHCO_3_ (100 mL) in the presence of ethylacetate (EtOAc). The phases were allowed to separate, small amounts of brine and DCM were added to aid separation. The aqueous phase was extracted once with EtOAc (100 mL), the organic layers were washed with H_2_O (2x 150 mL) and dried over MgSO_4_. After filtration the solvent was removed in vacuum to yield crude nelfinavir which was used without further purification in the following steps.

**Synthesis of (3*S*,4aS,8a*S*)-2-((2*R*,3*R*)-3-(3-(2-(3-(but-3-yn-1-yl)-3*H*-diazirin-3-yl)ethoxy)-2-methylbenzamido)-2-hydroxy-4-(phenylthio)butyl)-*N*-(*tert*-butyl)decahydroisoquinoline-3-carboxamide (SC441)**

Crude nelfinavir (1.0 eq., 794 mg, 1.40 mmol) was dissolved in DMF (10 mL), K_2_CO_3_ (1.1 eq., 213 mg, 1.54 mmol) was added and the suspension was warmed to 60 °C and protected from light by wrapping in aluminium foil. 3-(but-3-yn-1-yl)-3-(2-iodoethyl)-3*H*-diazirine (**1**, synthesized from ethylacetoacetate according to literature [[3](#_ENREF_3), [4](#_ENREF_4)]) (0.9 eq., 312 mg, 1.26 mmol) was dissolved in DMF (10 mL) and added over 3h at 60 °C, the reaction was kept at this temperature for 3h after the addition. The suspension was then carefully decanted and the solvent removed *in vacuo*. The crude product was purified by preparative reversed-phase HPLC (linear gradient 45→55% B, 10 min), the product fractions were concentrated and lyophilized to obtain SC-441·TFA as a white powder (89.6 mg, 0.112 mmol, 8.9%, based on **1**). ^1^H NMR (600 MHz, DMSO-d_6_): δ 8.90 (bs, 1H), 7.99 (s, 1H), 7.97 (d, *J* = 2.7 Hz, 1H), 7.12-7.08 (m, 4H), 6.98 (t, *J* = 7.0 Hz, 1H), 6.94 (t, *J* = 7.84 Hz, 1H), 6.76-6.73 (m, 2H), 3.87 (t, *J* = 8.2 Hz, 1H), 3.73-3.69 (m, 1H), 3.66 (bs, 1H), 3.60-3.59 (m, 2H), 3.18 (d, *J* = 11.3 Hz, 1H), 3.06- 3.04 (m, 1H), 2.98 (d, *J* = 13.1 Hz, 1H), 2.79-2.75 (m, 2H), 2.58 (s, 1H), 2.26 (m, 2H), 2.02 (s, 3H), 1.96-1-95 (m, 1H), 1.81 (td, *J* = 7.3, 2.5 Hz, 2H) 1.74-1.65 (m, 5H), 1.55-1.51 (m, 1H), 1.48-1.43 (m, 4H), 1.32-1.27 (m, 2H) 1.17- 1.10 (m, 3H), 0.99 (s, 9H). ^13^C NMR (151 MHz, DMSO-d_6_): δ 169.4, 167.1, 156.3, 138.5, 136.0, 129.0, 128.4, 126.2, 125.9, 123.5, 199.4, 112.1, 83.1, 71.8, 68.4, 58.6, 57.5, 52.2, 51.0, 33.7, 31.9, 31.8, 31.2, 30.4, 29.8, 28.4, 28.2, 27.1, 25.5, 24.5, 19.9, 12.7, 12.7. LC-MS (linear gradient 10% → 90% B, 0.1% TFA, 15 min): R_t_: 7.56 min, ESI-MS (m/z): 688.07 [M+H]^+^. HRMS: calculated for C_39_H_54_N_5_O_4_S*^+^* [M+H]*^+^* 688.38910; found 688.38938.

Alternatively, a portion of crude SC441 obtained using the conditions described (using 1.29 mmol crude nelfinavir) above was purified by flash column chromatography (SiO2, 0% → 1% → 2% MeOH/DCM) to obtain pre-purified SC441 which was used in the synthesis of SC451.

**Synthesis of (2*R*,3*R*)-3-(3-(2-(3-(but-3-yn-1-yl)-3*H*-diazirin-3-yl)ethoxy)-2-methylbenzamido)-1-((3*S*,4a*S*,8a*S*)-3-(*tert*-butylcarbamoyl)octahydroisoquinolin-2(1*H*)-yl)-4-(phenylthio)butan-2-yl pentanoate (SC451)**

Pre-purified SC441 (1.0 eq., 90 mg, 0.13 mmol) was dissolved in DMF (10mL) and the reaction flask wrapped in aluminium foil. 4-(dimethylamino)-pyridine (DMAP) (2.0 eq., 43 mg, 0.26 mmol) and valeric acid (2.0 eq., 29 µL, 0.26 mmol) were added and the mixture was cooled to 0 °C. Next, *N*-(3-dimethylaminopropyl)-*N*′-ethylcarbodiimide hydrochloride (EDC) (2.0 eq., 50 mg, 0.26 mmol) was added and the reaction mixture was allowed to warm to rt and stirred overnight. After 16h the reaction was checked by LC-MS, DMAP (2.0 eq., 43 mg, 0.26 mmol), valeric acid (2.0 eq., 29 µL, 0.26 mmol) and EDC (2.0 eq., 50 mg, 0.26 mmol) were added and the reaction mixture was stirred for another 20h at rt. After this time valeric acid (2.0 eq., 29 µL, 0.26 mmol) added and the reaction stirred for an additional 16h after which LC-MS analysis indicated a completed reaction.

The solvent was removed *in vacuo*. The crude product was purified by preparative reversed-phase HPLC (linear gradient 55→65% B, 10 min), the product fractions were concentrated and lyophilized to yield SC-451·TFA as a white powder (42.8 mg, 0.048 mmol, 37%). ^1^H NMR (500 MHz, DMSO-d_6_): δ 8.28 (bs, 1H), 7.45 (bs, 2H), 7.33-7.28 (m, 3H) 7.23-7.16 (m, 3H), 6.95 (d, *J* = 8.2 Hz, 1H), 6.84 (d, *J* = 7.5, 1H), 5.39 (bs, 1H), 4.53-4.48 (m, 1H), 3.85-3.78 (m, 3H), 3.68-3.57 (m, 2H), 3.15-3.02 (m, 2H), 2.83 (s, 1H), 2.34-2.26 (m, 3H), 2.20 (s, 3H), 2.04 (td, *J* = 7.4, 2.7 Hz, 2H), 1.94-1.85 (m, 5H), 1.71-1.64 (m, 3H), 1.54-1.47 (m, 6H), 1.40-1.35 (m, 2H), 1.31-1.24 (m, 6H), 1.24-1.12 (m, 9H), 0.85 (t, *J* = 7.3 Hz, 3H). ^13^C NMR (126 MHz, DMSO-d_6_): δ172.4, 169.2, 156.4, 147.8, 139.0, 129.0, 128.4, 127.8, 127.6, 126.7, 126.4, 123.2, 119.0, 111.9, 83.2, 71.8, 70.7, 62.8, 49.0, 33.3, 31.9, 31.8, 31.3, 28.1, 27.2, 26.2, 25.8, 24.9, 21.7, 20.2, 20.1, 13.7, 12.7, 12.5. LC-MS (linear gradient 10% → 90% B, 0.1% TFA, 15 min): R_t_: 8.43 min, ESI-MS (m/z): 772.27 [M+H]^+^. HRMS: calculated for C_44_H_62_N_5_O_5_S^+^ [M+H]^+^ 772.44662; found 772.44691.

**SUPPLEMENTAL FIGURES**

**Supplemental Figure SI1:** Time response of ABCB1 inhibition evaluated by MTG efflux after treatment with 10 μM concentration of indicated compounds. Significant values <0.05 are marked with *.





**Supplemental Figure SI2:** Chemical structure of truncated (SC451) and active (SC441) NFV-based compounds.





**Supplemental Figure SI3:** Dose response curves of carfilzomib co-treated with NFV (10 μM) LPV (10 μM) and PK11195 (12.5, 25, 50 μM) in AMO-CFZ cells. Corresponding IC_50_ values are presented in Supplemental Table SI8.


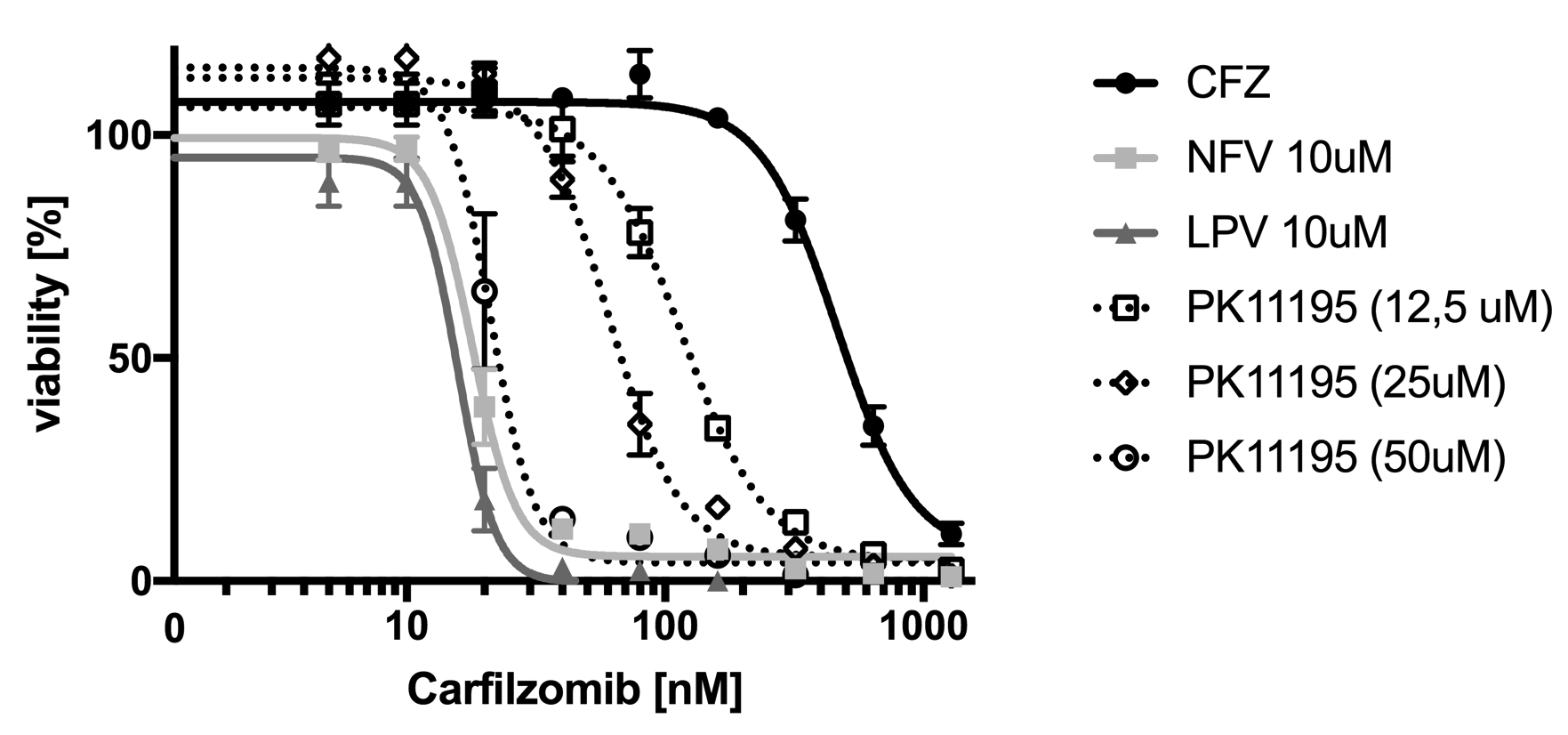


**SUPPLEMENTAL TABLES**

**Supplemental Table SI1:** List of patients’ bone marrow plasma cells (BMPC) and circulating peripheral blood plasma cells (PB-PC) and classification according to the Total Therapy that was used.

| Total Therapy | BMPC of newly diagnosed MM patients | Circulating PB-PC |
| --- | --- | --- |
| TT2- | 169 | 1 |
| TT2+ | 176 | 4 |
| TT3a | 274 | 3 |
| TT3b |  | 24 |
| TT4 |  | 2 |
| TT5 |  | 7 |
| TT6 |  | 3 |

**Supplemental Table SI2:** IC_50_ values for bortezomib (BTZ) and carfilzomib (CFZ) in AMO-1 sensitive and resistant cells (AMO-BTZ, AMO-CFZ) accompanying Figure 2B.

| IC_50_ | BTZ [nM] | CFZ [nM] |
| --- | --- | --- |
| AMO-1 | 6.3 (±0.3) | 4.8 (±0.1) |
| AMO-BTZ | 1342.5 (±62.5) | 67.2 (±7.2) |
| AMO-CFZ | 89.3 (±3) | 891.9 (±37.9) |

**Supplemental Table SI3:** IC_50_ values for carfilzomib (CFZ) in AMO-CFZ clones with (#1) or without ABCB1 (#7, #8, #14) accompanying Figure 3B. Significant differences (p<0.05) of IC_50_ values are marked (*).

| IC_50_ | #1 | #7 | #8 | #14 |
| --- | --- | --- | --- | --- |
| CFZ [nM] | 826.8 (±45.8) | 115.4 (±5)* | 423.5 (±5.9)* | 270.2 (±5)* |

**Supplemental Table SI4:** Comparison of IC_50_ values for drugs approved for MM treatment or in clinical development in AMO-CFZ adapted cell line with upregulated ABCB1 (#1) and depleted ABCB1 (#7), and their ratio. Significant differences (p<0.05) of IC_50_ values are marked (*).

| IC_50_ (nM) | #1 | #7 | #1/#7 |
| --- | --- | --- | --- |
| Panobinostat | 220.2 (±7.4) | 49.5 (±2.8)* | 4.4 |
| Cyclophosphamide | 16.1 (±0.4) | 10.1 (±0.6)* | 1.6 |
| Daunorubicin (μM) | 21.3 (±6.41) | 4.41 (±0.53)* | 4.8 |
| Lenalidomide (μM) | 1747.8 (±22.4) | 672.4 (±48.8)* | 2.6 |
| Bortezomib | 69.3 (±1.3) | 26.8 (±1.5)* | 2.6 |
| Carfilzomib | 613.3 (±15.6) | 78.5 (±9.4)* | 7.8 |
| Delanzomib | 293.9 (±19.8) | 80.5 (±4.2)* | 3.7 |
| Ixazomib | 692.5 (±46.8) | 345.4 (±15.7)* | 2.0 |
| Oprozomib | 1469.8 (±37.8) | 220.6 (±34.3)* | 6.7 |
| Marizomib | 61.4 (±0.9) | 32.9 (±0.8)* | 1.9 |

**Supplemental Table SI5:** IC_50_ values for carfilzomib (CFZ) alone or in combination with nelfinavir (NFV), NFV truncated analogue SC451 or functional analogue SC441 in AMO-CFZ cells accompanying Figure 6B. Significant differences (p<0.05) of IC_50_ values are marked (*).

| IC_50_ | AMO-CFZ |
| --- | --- |
| CFZ [nM] | 506.9 (±0.3) |
| CFZ [nM]+SC451 [10 µM] | 364.8 (±62.5)* |
| CFZ [nM]+SC441 [10 µM] | 5.0 (±3)* |

**Supplemental Table SI6:** IC_50_ values for A) carfilzomib (CFZ), co-treatment with nelfinavir (NFV) or lopinavir (LPV), and the ratio of CFZ vs CFZ+NFV or CFZ vs CFZ+LPV; B) CFZ and co-treatment with verapamil (VPM) or reserpine (RSP), and the ratio of CFZ vs CFZ+VPM or CFZ vs CFZ+RSP in AMO-CFZ clones with ABCB1 (#1) or with depleted ABCB1 (#7) accompanying Figure 6C. Significant differences (p<0.05) of IC_50_ are marked (*).

**A)**

| IC_50_ | #1 | #7 |
| --- | --- | --- |
| CFZ [nM] | 795.6 (±24.8) | 127.2 (±2.9)* |
| CFZ [nM]+NFV [10 uM] | 38.6 (±2.3) | 8.1 (±0.6)* |
| CFZ [nM]+LPV [10 uM] | 20 (±1.7) | 9.3 (±1.1)* |
| Ratio CFZ/ CFZ+NFV | 20.6 | 15.7 |
| Ratio CFZ/ CFZ+LPV | 39.8 | 13.7 |

**B)**

| IC_50_ | #1 | #7 |
| --- | --- | --- |
| CFZ [nM] | 823.6 (±33.1) | 119.2 (±5.7)* |
| CFZ [nM]+VPM [10 uM] | 49.23 (±1.6) | 10.74 (±0.5)* |
| CFZ [nM]+RSP [10 uM] | 14.21 (±0.7) | 9.3 (±1.1)* |
| Ratio CFZ/ CFZ+VPM | 16.8 | 11.09 |
| Ratio CFZ/ CFZ+RSP | 57.9 | 13.6 |

**Supplemental Table SI7:** IC_50_ values for already approved PI (BTZ=bortezomib, CFZ=carfilzomib, IXA=ixazomib) or PI in advanced clinical development (DLZ=delanzomib, Opro=oprozomib, PR957=β5 specific inhibitor, MRZ=marizomib) and co-treatement with nelfinavir (NFV) or lopinavir (LPV) in A) AMO-1, B) AMO-BTZ, C) AMO-CFZ cells accompanying Figure 7. Significant differences (p<0.05) of IC_50_ between PI treatment alone or in combinations are marked (*).

**A)**

**AMO-1**

| IC_50_ | PI [nM] | PI [nM]+NFV [10 µM] | PI [nM]+LPV [10 µM] |
| --- | --- | --- | --- |
| BTZ | 6.3 (±0.3) | 5.6 (±0.1)* | 5.1 (±0.1)* |
| CFZ | 2.4 (±0.1) | 1.0 (±0.1) | 1.0 (±0.1)* |
| DLZ | 11.0 (±1.6) | 10.5 (±0.7) | 10.1 (±0.5) |
| IXA | 33.8 (±4.4) | 26.0 (±2.2)* | 28.2 (±2.7) |
| Opro | 16.2 (±0.4) | 12.6 (±0.5)* | 10.9 (±0.5)* |
| PR957 | 43.3 (±2.8) | 32.7 (±2.7)* | 52.4 (±8.4) |
| MRZ | 30.2 (±1.9) | 13.7 (±1.8)* | 9.1 (±1.8)* |

**B)**

**AMO-BTZ**

| IC_50_ | PI [nM] | PI [nM]+NFV [10 µM] | PI [nM]+LPV [10 µM] |
| --- | --- | --- | --- |
| BTZ | 1359.0 (±7) | 725.2 (±11.1)* | 615.9 (±23.6)* |
| CFZ | 67.2 (±7.2) | 24.7 (±1.4)* | 20.3 (±0.2)* |
| DLZ | >1280 | >1280 | >1280 |
| IXA | >1280 | >1280 | >1280 |
| Opro | 733.9 (±11.6) | 567.9 (±41.1)* | 427.7 (±9.3)* |
| PR957 | 2254.3 (±430.5) | 1780.5 (±545.2) | 1605.8 (±199.9)* |
| MRZ | 629.0 (±72) | 407.2 (±89.6)* | 270.7 (±61.9)* |

**C)**

**AMO-CFZ**

| IC_50_ | PI [nM] | PI [nM]+NFV [10 µM] | PI [nM]+LPV [10 µM] |
| --- | --- | --- | --- |
| BTZ | 155.7 (±3.5) | 34.7 (±0.8)* | 34.7 (±3.3)* |
| CFZ | 564.4 (±15.6) | 7.1 (±0.5)* | 4.7 (±0.4)* |
| DLZ | 1044.9 (±50.7) | 166.0 (±5.8)* | 161.0 (±17.1)* |
| IXA | 1285.0 (±25.3) | 688.5 (±64.2)* | 651.1 (±75.8)* |
| Opro | 1318.3 (±4.6) | 143.7 (±8.1)* | 101.5 (±9.4)* |
| PR957 | <1280 | 664.5 (±43.2)* | 513.3 (±61.6)* |
| MRZ | 92.8 (±9.9) | 68.8 (±9.6)* | 68.0 (±12.3)* |

**Supplemental Table SI8:** IC_50_ values for carfilzomib (CFZ) in AMO-CFZ accompanying Supplemental Figure SI3. Significant differences (p<0.05) of IC_50_ values are marked (*).

| IC_50_ | PI [nM] |
| --- | --- |
| CFZ | 462.7 (±41.6) |
| CFZ+NFV [10uM] | 17.97 (±6.4) |
| CFZ+LPV [10uM] | 15.79 (±7.1) |
| CFZ+PK11195 [12.5uM] | 116.1 (±25.3) |
| CFZ+PK11195 [25uM] | 60.69 (±8.3) |
| CFZ+PK11195 [50uM] | 21.03 (±1.9) |

**REFERENCES**

1. Dantuma, N.P., et al., *Short-lived green fluorescent proteins for quantifying ubiquitin/proteasome-dependent proteolysis in living cells.* Nat Biotechnol, 2000. **18**(5): p. 538-43.

2. Greiner, A., et al., *Activity and subcellular distribution of cathepsins in primary human monocytes.* J Leukoc Biol, 2003. **73**(2): p. 235-42.

3. Li, Z., et al., *Design and synthesis of minimalist terminal alkyne-containing diazirine photo-crosslinkers and their incorporation into kinase inhibitors for cell- and tissue-based proteome profiling.* Angew Chem Int Ed Engl, 2013. **52**(33): p. 8551-6.

4. Hayakawa, K., et al., *Novel bicycloannulation via tandem vinylation and intramolecular Diels-Alder reaction of five-membered heterocycles: a new approach to construction of psoralen and azapsoralen.* Journal of the American Chemical Society, 1984. **106**(22): p. 6735-6740.
